# Giving Made Easy

**Published:** 1870-04

**Authors:** 


### GIVING MADE EASY.

GIVE often. That tells the whole story. So it is in many of the habits of life. If you want to preach easily, preach often, thus verifying the old saw, "Practice makes perfect." The man who writes a dozen letters a day, will accomplish it with half the effort of one who writes but one in a week, or month, or year.

### GOING TO CHURCH.

Regular church-goers find no trouble in going to meeting every time the bell rings, for it is done as a matter of course, their mind is made up to it, and they would feel " out of sorts " if they missed a single time. Even disagreeable things are made pleasurable, delightful, by persistent practice. The first drink of whiskey always causes a wry face, and yet, according to the story, an enthusiastic individual, after a year's practice, wished his throat was a mile long, it felt so good in its way down.

If a man excuses himself from going to church on the Sabbath after having been conscientiously trained to be a regular attendant, his conscience will trouble him the first time, but less and less after each successive omission, until after a while, it is no trouble at all for him to stay at home, and between its being too warm or too cold, too wet or too dusty, he seldom enters a church door, and he never enters the kingdom o heaven, for " the door is shut." Let every man then watch over his habits, cultivate those which are good, break off from those which in the end destroy both body and soul.

